# Supplementary material for: The Nine-Item Internet Gaming Disorder Scale (IGDS9-SF): Its Psychometric Properties among Sri Lankan Students and Measurement Invariance across Sri Lanka, Turkey, Australia, and the USA
Source: Healthcare (Basel). 2022 Mar 7;10(3):490. doi: 10.3390/healthcare10030490 (PMC8953588; doi:10.3390/healthcare10030490)
Supplement: Supplementary file 1 [file healthcare-10-00490-s001.zip › healthcare-1611378-supplementary.pdf]

Table S1. Invariance of the IGD-SF9 across different groups among university students from Sri Lanka

Groups	Invariance Levels	χ^2	df	*p*	$\Delta\chi^2$	Δdf	$p(\Delta\chi^2)$	CFI	ΔCFI	TLI	ΔTLI	RMSEA	ΔRMSEA	SRMR
Gender	Configural	76.766	54	0.023				.963		.951		.036		0.0390
	Metric	83.651	62	.035	6.885	8	0.549	.965	-0.002	.959	-0.008	.033	0.003	0.0463
	Strong	85.846	63	.029	2.195	1	.138	.963	0.002	.958	0.001	.034	-0.001	0.0571
	Strict	120.844	72	.000	34.997	9	.001	.921	**0.042**	.921	**0.037**	.046	-0.012	0.0464
Ethnicity	Configural	84.134	54	.005				.954		.939		.042		0.0430
	Metric	90.936	62	.010	6.802	8	0.558	.956	-0.002	.949	0.010	.038	0.004	0.0436
	Strong	92.457	63	.009	1.522	1	.217	.955	0.001	.949	0.000	.038	0.000	0.0447
	Strict	98.823	72	.020	6.365	9	.703	.959	-0.004	.959	0.010	.034	0.004	0.0453
Language of the questionnaire	Configural	83.561	54	.006				.956		.942		.041		0.0520
	Metric	86.519	62	.022	2.959	8	0.937	.964	-0.008	.958	-0.016	.035	0.006	0.0529
	Strong	91.431	63	.011	4.912	1	.027	.958	0.006	.952	0.006	.038	-0.003	0.0661
	Strict	101.047	72	.014	9.616	9	.382	.957	0.001	.957	-0.005	.036	0.002	0.0685
Academic major	Configural	181.809	144	.018				.945		.945		.029		0.0846
	Metric	197.609	152	.008	15.800	8	0.045	.934	0.011	.937	0.008	.031	-0.003	.0920
	Strong	200.649	153	.006	3.039	1	0.081	.931	0.003	.935	0.002	.032	-0.001	.0987
	Strict	230.161	162	.000	29.512	9	.001	.901	0.030	.912	0.023	.037	-0.005	.1035
Household income	Configural	124.237	99	0.044				.963		.960		.028		0.0680
	Metric	147.471	107	.006	23.234	8	0.003	.941	**0.022**	.940	**0.020**	.034	0.008	0.0843
	Strong	147.546	108	.007	0.075	1	.785	.942	-0.001	.942	-0.002	.034	-0.004	0.0862

	Strict	168.628	117	.001	21.082	9	.012	.925	0.017	.930	0.012	.037	-0.003	0.0741
Device	Configural	65.723	54	.132				.982		.976		.026		0.0392
	Metric	76.295	62	.105	10.572	8	0.227	.978	0.004	.975	0.001	.027	-0.001	0.0430
	Strong	76.567	63	.117	0.272	1	.602	.980	-0.002	.977	-0.002	.026	0.001	0.0430
	Strict	83.104	72	.174	6.537	9	.685	.983	-0.003	.983	-0.006	.022	0.004	0.0448
Game type	Configural	150.916	99	.001				.923		.916		.041		0.0782
	Metric	158.040	107	.001	7.124	8	0.523	.925	-0.002	.924	-0.008	.039	0.002	0.0862
	Strong	158.178	108	.001	0.138	1	.710	.926	-0.001	.926	-0.002	.038	0.001	0.0860
	Strict	186.206	117	.000	28.028	9	.001	.898	**0.028**	.906	**0.020**	.043	-0.005	**0.0931**

χ^2, chi-square; df, degrees of freedom; CFI, comparative fit index; TLI, Tucker–Lewis index; RMSEA, root mean square error of approximation; SRMR, standardized root mean residual.
